# Supplementary figures and images for: MeCP2 gates spatial learning-induced alternative splicing events in the mouse hippocampus
Source: Mol Brain. 2020 Nov 17;13:156. doi: 10.1186/s13041-020-00695-1 (PMC7672966; doi:10.1186/s13041-020-00695-1)

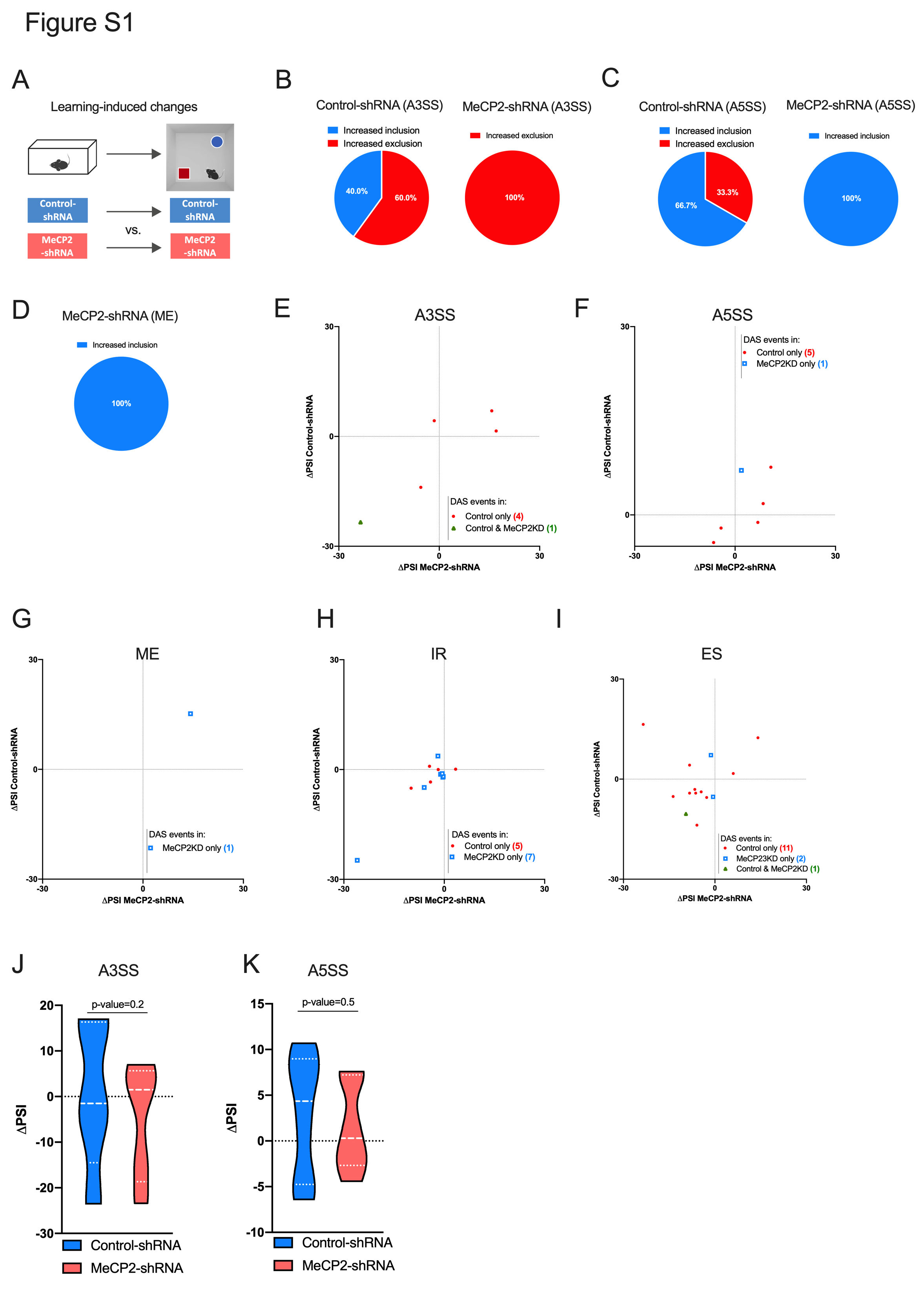

Supplement: Supplementary file 3 — Additional file 3: Figure S1. Alternative splicing event-specific changes in MeCP2 knockdown mice upon spatial learning. A) Schematic representation of the comparisons used. B-D) Pie charts showing the proportion of learning-induced inclusion and exclusion events for (B) alternative 3′ splice sites (A3SS), (C) alternative 5′ splice (A5SS) and (D) mutually exclusive exons (ME) in Control-shRNA and MeCP2-shRNA mice. E-I) Scatter plots showing changes in (E) A3SS, (F) A5SS, (G) ME, (H) intron retention (IR) and (I) exon skipping (ES) events in Control-shRNA (Control) and MeCP2-shRNA (MeCP2KD) mice upon learning. Red dots and blue squares represent alternative splicing events occurred in either Control or MeCP2-knock-down (MeCP2KD) (q-value < 0.05), respectively. Green triangles represent alternative splicing events that occurred in both conditions (q-value < 0.05). J-L) Violin plots showing the ΔPSI distribution of A3SS (J), A5SS (K) events in Control-shRNA and MeCP2-shRNA hippocampi after learning. The P-values are based on paired two-tailed Student’s t test or Wilcoxon test and are indicated at the top of each panel. ΔPSI: delta “percent spliced in”. [file 13041_2020_695_MOESM3_ESM.tiff]

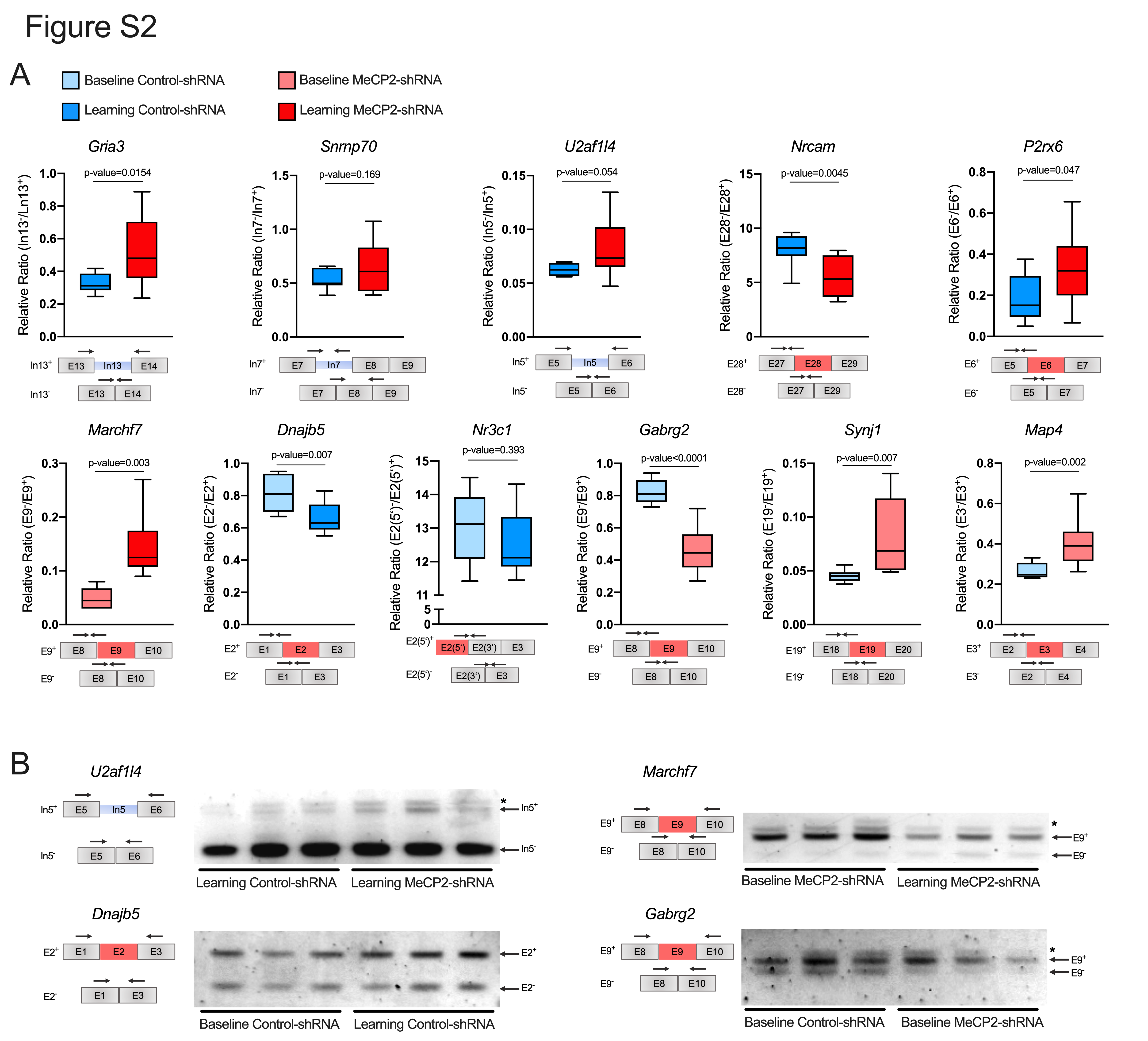

Supplement: Supplementary file 5 — Additional file 5: Figure S2. Validation of genes that underwent differential alternative splicing. (A) Quantitative RT-PCR for differential alternative splicing events. Box plots show the relative ratio of retained vs. excised introns (IR) or included vs. excluded exons (ES, A5SS) between conditions (N = 8–10). The diagram below each individual gene shows the two possible isoforms (included/excluded exon or intron in red or blue, respectively) with arrows indicating the location of the primers used (Additional file 1: Table S1). The P-values are based on unpaired two-tailed Student’s t test or Mann–Whitney test indicated at the top of each panel. (B) Semiquantitative RT-PCR for genes that underwent differential alternative splicing. Gel images show the retained vs. spliced isoform intensity. Diagram next to gene name shows the location of the primers used (Additional file 1: Table S1). Three biological replicates are shown per condition. *refers to unspecific PCR products based on size prediction. [file 13041_2020_695_MOESM5_ESM.tiff]

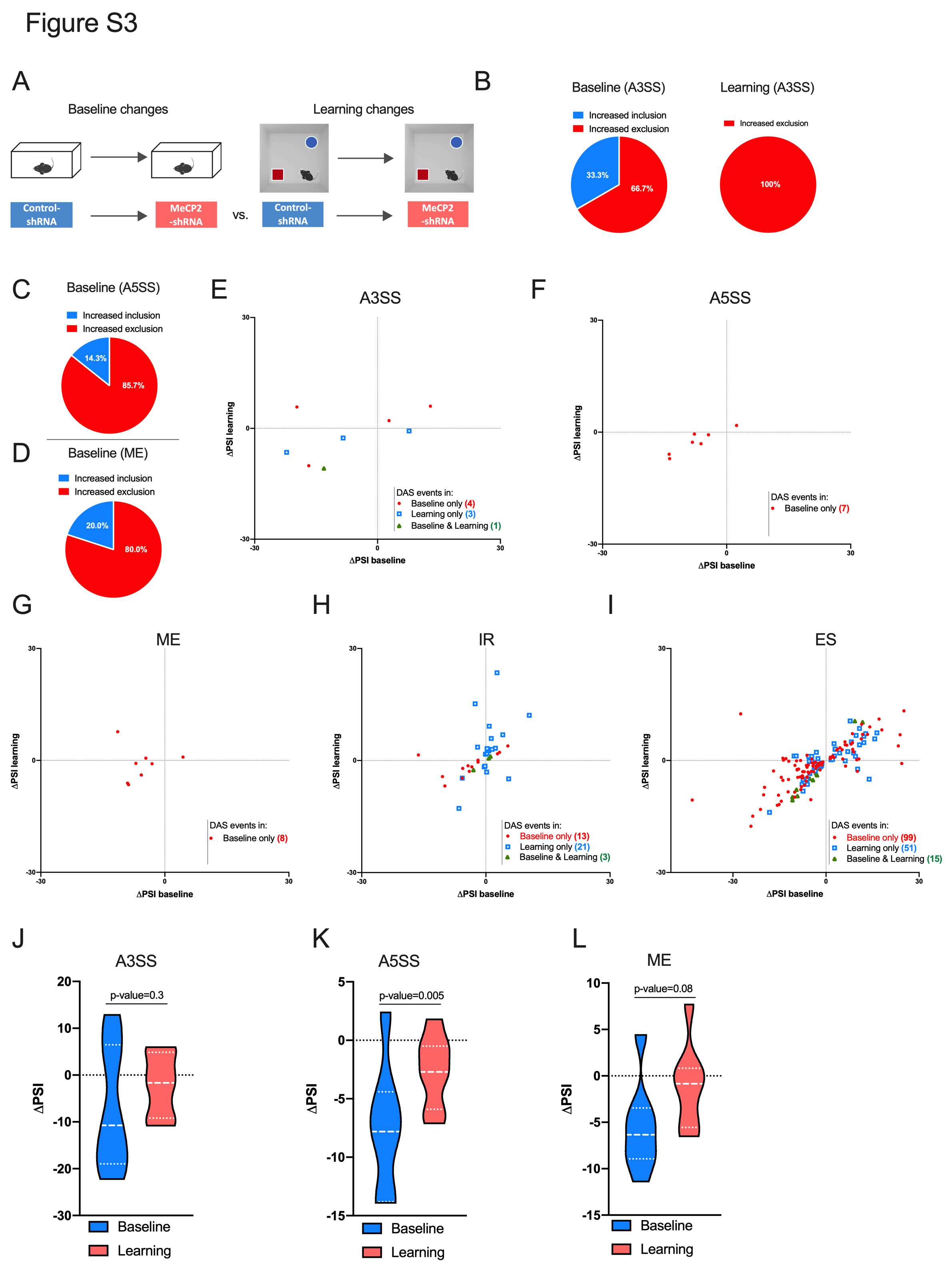

Supplement: Supplementary file 9 — Additional file 9: Figure S3. Alternative splicing event-specific changes in MeCP2 knock-down mice during baseline and learning state conditions. A) Schematic representation of the comparisons used. B-D) Pie charts showing the proportion of inclusion and exclusion events for (B) alternative 3′ splice sites (A3SS), and (C) alternative 5′ splice (A5SS) and (D) mutually exclusive exons (ME) in MeCP2-shRNA mice during baseline and learning-state conditions. Note that no ME changes were detected by MeCP2 knockdown in learning-state. E-I) Scatter plots showing changes in (E) A3SS, (F) A5SS, (G) ME, (H) intron retention (IR) and (I) exon skipping (ES) events in home-cage (Baseline) and learning state (Learning) conditions in MeCP2-shRNA mice compared to the controls. Red dots and blue squares represent alternative splicing events occurred in either baseline or learning conditions (q-value < 0.05), respectively. Green triangles represent alternative splicing events that occurred in both conditions (q-value < 0.05). J-L) Violin plots showing the ΔPSI distribution of (J) A3SS, (K) A5SS and (L) ME events in baseline and learning state conditions in the dorsal hippocampi of MeCP2-shRNA mice. The P-values are based on paired two-tailed Student’s t test or Wilcoxon test. and are indicated at the top of each panel. ΔPSI: delta “percent spliced in”. [file 13041_2020_695_MOESM9_ESM.tiff]
